# Policy implications of the potential use of a novel vaccine to prevent infection with *Schistosoma mansoni* with or without mass drug administration

**DOI:** 10.1016/j.vaccine.2020.04.078

**Published:** 2020-06-09

**Authors:** Klodeta Kura, Benjamin S. Collyer, Jaspreet Toor, James E. Truscott, T. Deirdre Hollingsworth, Matt J. Keeling, Roy M. Anderson

**Affiliations:** a London Centre for Neglected Tropical Disease Research, London, United Kingdom; b Department of Infectious Disease Epidemiology, School of Public Health, Faculty of Medicine, St Mary’s Campus, Imperial College London, London, United Kingdom.; c MRC Centre for Global Infectious Disease Analysis, United Kingdom; d Mathematics Institute, University of Warwick, United Kingdom; e School of Life Sciences, University of Warwick, United Kingdom; f The DeWorm3 Project, The Natural History Museum of London, London, United Kingdom; g Big Data Institute, Li Ka Shing Centre for Health Information and Discovery, University of Oxford, Oxford OX3 7LF, United Kingdom

**Keywords:** Schistosomiasis, Vaccine, Modelling, Morbidity, Elimination, Control

## Abstract

Schistosomiasis is one of the most important neglected tropical diseases (NTDs) affecting millions of people in 79 different countries. The World Health Organization (WHO) has specified two control goals to be achieved by 2020 and 2025 - morbidity control and elimination as a public health problem (EPHP). Mass drug administration (MDA) is the main method for schistosomiasis control but it has sometimes proved difficult to both secure adequate supplies of the most efficacious drug praziquantel to treat the millions infected either annually or biannually, and to achieve high treatment coverage in targeted communities in regions of endemic infection. The development of alternative control methods remains a priority.

In this paper, using stochastic individual-based models, we analyze whether the addition of a novel vaccine alone or in combination with drug treatment, is a more effective control strategy, in terms of achieving the WHO goals, as well as the time and costs to achieve these goals when compared to MDA alone. The key objective of our analyses is to help facilitate decision making for moving a promising candidate vaccine through the phase I, II and III trials in humans to a final product for use in resource poor settings.

We find that in low to moderate transmission settings, both vaccination and MDA are highly likely to achieve the WHO goals within 15 years and are likely to be cost-effective. In high transmission settings, MDA alone is unable to achieve the goals, whereas vaccination is able to achieve both goals in combination with MDA. In these settings Vaccination is cost-effective, even for short duration vaccines, so long as vaccination costs up to US$7.60 per full course of vaccination. The public health value of the vaccine depends on the duration of vaccine protection, the baseline prevalence prior to vaccination and the WHO goal.

## 1 Introduction

The World Health Organization (WHO) has noted that schistosomiasis is only second to malaria as the most devastating parasitic disease in terms of socioeconomic importance and public health impact [1]. According to the latest Global Burden of Disease estimates, more than 229 million people are infected with *Schistosoma* species, including 124 million school-aged children - mostly in sub-Saharan Africa [2]. Approximately 5–10% of individuals infected by *Schistosoma mansoni* suffer from severe hepatic periportal fibrosis which is the leading cause of death from schistosomiasis, and can lead to portal hypertension, hepatosplenomegaly and esophageal varices [3].

Morbidity control and elimination as a public health problem (EPHP) is defined by WHO as prevalence of heavy-intensity infection in school-aged children (SAC, ages 5–14) ≤5% and ≤1% respectively [4]. The WHO treatment guidelines are primarily based on treating SAC only who typically harbor most infection (a target MDA coverage of 75% is recommended), and those at high risk of infection via mass drug administration (MDA) of the target groups using praziquantel (PZQ) [2]. Despite the burden of disease in pre-school-aged children (PSAC), the use of PZQ in this age group is limited because a pediatric indication of PZQ is not available at present. A new formulation may make treating very young children possible in the near future [5].

In this work we use the WHO-recommended treatment frequencies, based on the pre-treatment baseline prevalence of infection in SAC [6]. We administer MDA once a year in high transmission settings (≥50% baseline prevalence among SAC), once every two years in moderate transmission settings (10–50% baseline prevalence among SAC) and once every 3 years in low transmission settings (<10% baseline prevalence among SAC).

Previous analyses based on mathematical models of transmission and MDA impact suggest that by following the WHO recommended treatment coverage and frequency for MDA, the WHO goals in low to moderate transmission settings can be achieved, but it is highly unlikely that the goals will be achieved in high transmission settings [7], [8], [9], [10], [11], [12], [13], [14]. Furthermore, achieving high coverage levels of 75% in SAC required to reach the morbidity targets will be difficult given past experience. The current MDA coverage level reported by WHO for all endemic regions (international scale) is 61.2% of SAC and 18.2% of adults [2], [15].

Other issues with schistosome targeted MDA programs include: (i) drug treatment does not prevent re-infection due to the absence of protective acquired immunity and therefore repeated drug administration is necessary; (ii) the emergence of drug resistance is a constant threat as PZQ has been used for over three decades [16], [17]; (iii) a substantial infrastructure is required to ensure the drug is supplied regularly within an acceptable time frame; (iv) evidence is emerging of hybridization of human schistosome species with those that typically infect livestock such that in some regions the infection may become zoonotic with all the associated problems of eliminating infection in humans in the presence of an animal reservoir [18]; (v) limited supplies of the main drug PZQ in settings wishing to implement community wide treatment; (vi) high spatial heterogeneity in the prevalence of infection which requires high resolution mapping of the prevalence of infection to ensure the right communities are treated; and (vii) the necessity to ensure that MDA coverage is high and that individual compliance to treatment is good such that the proportion of persistent non-compliers to treatment is very low to ensure they do not provide a persistent reservoir of infection [19].

Schistosomiasis is considered to be one of the 10 diseases where vaccines are urgently required to be part of treatment programs to reduce the probability of reinfection and eliminate the parasite in endemic regions, particularly in high transmission settings [20].

At present there is no vaccine for use in humans that can protect against schistosome infection. However, there are various candidate schistosomiasis vaccines in different stages of clinical trials [21]. Monovalent recombinant protein Sm14 with GLA-SE adjuvant has successfully completed phase I and phase II of clinical trials with phase IIb underway in school-age children [21], [22] Bilvax-monovalent recombinant protein Sh28GST is another candidate vaccine, against *S. haematobium*, that has successfully completed phase I and phase II clinical trials. A phase III randomized controlled trial was completed in Senegal (children only) to assess the safety and efficacy of the vaccine. Published results show that the vaccine induced a consistent immune response, but the efficacy endpoint was not achieved [21], [23], [24]. We note here, that these two vaccines target only one specific species.

Sm-p80 is another promising candidate vaccine, considered as a leading candidate as it can target three species, *S. mansoni, S. haematobium and S. japonicum* [25]*.* Sm-p80 is a large subunit of the S. mansoni calpain protein gene. This candidate vaccine has been tested for the prophylactic and anti-fecundity efficacy in various vaccine formulations and delivery approaches, in three animal models (mouse, hamster and baboon). It is shown that this vaccine is effective against different stages of the parasite’s life cycle and most importantly, Sm-p80 specific IgE has not been detected in humans in endemic areas such as Africa and South America. This implies that there might be a low probability of hypersensitivity reaction following vaccination in humans [25], [26].

Pre-clinical experimental studies, against *Schistosoma mansoni* infection in a baboon model (*Papio anubis*) have produced very encouraging results. The study showed greatly reduced female worm establishment (by 93.45%) and tissue egg load (by 91.35%). Another important factor is the duration of vaccine protection and these experimental results suggest a 5–8 year duration of protection [27], [28]. A phase I clinical trial, funded by the National Institutes of Health, is underway in the United States.

Evaluating the potential community-level impact of a schistosome vaccine to protect against the disease schistosomiasis is required to quantitatively assess the benefits arising from what will be a very large investment to get the candidate vaccine through the development cycle. In such analyses, the cost per vaccine dose (or short course of doses) and the manner in which the vaccine is going to be used, either as a stand-alone intervention or in combination with other interventions such as mass drug administration, are of utmost importance.

This paper presents results based on epidemiological and mathematical analyses of the potential impact of such a vaccine, used either alone or in combination with MDA. The paper also details a comparison of the cost-effectiveness of each intervention. The aim is to provide policy insights that may help in decision making during the various phases of clinical development about the cost effectiveness of a vaccine with defined properties. Cost-effectiveness analyses with clearly defined morbidity and mortality benefits over given time spans, taking account of different cost estimates of each dose, or a short course of doses, are also essential for impact assessment.

## 2 Methods

Individual-based stochastic models of parasite transmission and control intervention impact have been developed independently by Imperial College London (ICL) [11], [14], [29], [30] and b) the University of Warwick (UOW) [31]. Both models produce very similar results for defined parameter sets (see Figure S.1-S.5). The parameters used in this study are outlined in Table S6. In both models we assume an ideal case perfect vaccine where the rate of infection and the rate of egg production are reduced by 100% (an assumption which is in good agreement with the baboon Sm-p80 experiments described earlier), for a variety of population or age-group coverage levels (where coverage is assumed to be at random at each round of treatment-i.e. no systematic non-adherence to MDA and/or vaccination) and vaccination uptake is given at a defined age in all scenarios.

### 2.1 Description of control strategies

Experimental results have demonstrated that the Sm-p80 vaccine has a duration of protection between 5 and 8 years in animal models [27]. There is no data on the duration of protection in humans, so our analysis uses three different protection durations, five years, ten years and an ideal case where the vaccine provides protection for 20 years post vaccination (or a short course of vaccination). The following six treatment strategies are examined.

1. Mass drug administration (MDA using PZQ) alone

This scenario addresses the question of whether the current efforts are going to lead to morbidity control or EPHP, and over what timescale. The following variations have been analyzed: MDA given to SAC only with treatment coverage of (i) 75% (WHO recommended), (ii) 60% or (iii) 40%. The treatment frequencies are based on SAC baseline prevalences as defined by WHO.

2. Cohort Immunization

This scenario examines the questions of whether, and under which conditions, a vaccine used alone achieves the goals of morbidity control or EPHP. For routine vaccination, six scenarios were simulated (shown in Table 1).

3. Cohort immunization with a catch-up vaccination campaign.

**Table 1:** Coverage levels (taken from within the ranges found in the literature for HPV and DTP [32], [33], [34], [35]) for the human host population for each treatment strategy examined in the simulations.

20-year average duration of vaccine protection	10-year average duration of vaccine protection	5-year average duration of vaccine protection [28]
Vaccinate once at age 1 yr (85% coverage)	Vaccinate twice at ages 1yrs and 11yrs (85% and 70% coverage, respectively)	Vaccinate three times at ages 1 yr, 6yrs and 11yrs (85%, 60% and 70% coverage, respectively)
Vaccinate once at age 5yrs (60% coverage)	Vaccinate twice at ages 5yrs and 15yrs (60% and 45% coverage, respectively)	Vaccinate three times at ages 5yrs, 10yrs and 15yrs (60%, 70% and 45% coverage, respectively)

This scenario includes a round of immunizing SAC, with coverages described in Table 1, before reverting to the same schedule as cohort immunization after year 1. The purpose of the catch-up is to have high coverage of the SAC age range from the beginning of the intervention.

4. Immunization of whole age groups.

An alternative to immunizing children in cohorts is to target larger groups of the population with a lower frequency. The following situations have been simulated: immunize annually, every two years and every five years the SAC or the whole community with coverage levels as described in Table 1

5. Cohort immunization + MDA.

This scenario examines the question of whether the addition of a vaccine (stand-alone or in combination with PZQ) is a more effective approach - in terms of a desired impact and time to achieve the impact - than MDA alone. The MDA and immunization program are implemented concurrently, with duration of protection and coverage levels from the scenarios described in use cases 1 to 4.

6. Cohort immunization with a catch-up campaign and MDA.

This scenario assesses the impact of the immunization of SAC in year 0–1, before reverting to the same schedule as Cohort immunization plus MDA after year 1.

### 2.2 Epidemiological analysis

Each of these treatment strategies is evaluated for low, moderate and high transmission settings (as defined by WHO and detailed in the Introduction). At 15 years post initiation, we calculate the probability of reaching the WHO morbidity and EPHP (Elimination as a Public Health Problem) goals, by evaluating SAC heavy-intensity infection prevalence (≤5% heavy-intensity infection in SAC for the morbidity goal and ≤ 1% heavy-intensity infection in SAC for the EPHP goal).

### 2.3 Cost-Effectiveness analysis

Cost-effectiveness calculations requires both a quantification of the health benefits of a given treatment program as well as the associated economic costs. To measure the benefit of applying an intervention, we count the total number of days that each individual has a heavy intensity infection (HII) and record the total high intensity infection years averted over a 30-year time period, relative to the baseline of no intervention. Heavy intensity infections have been chosen as our measure because this is where most of the negative health and societal effects occur.

The assumed costs of MDA, which are composed of the cost of praziquantel and costs relating to the delivery and administration of the drug to affected communities, are outlined in Table S5. The costs related to MDA delivery were assumed to be dependent on how many were targeted for the treatment and not the number subsequently treated. The total cost related to the PZQ tablets was directly dependent on how many were treated (Table S5). In the absence of a manufactured product with known storage requirements on shelf life expectancy, the costs of the vaccination program are unknown. Analyses have therefore been performed using three different costs of US$3, US$6 and US$12 per full course of vaccination per person – i.e. we consider the cost of vaccination as the cost the total number of doses needed to achieve immunity and is not the per-dose price. This provides a more precise way of comparing vaccines that may require different number of doses. These three costs cover a range consistent with current GAVI Alliance vaccine program costs, and are intended to include the cost of the vaccine, the supply chain and of service delivery [36].

We quantify the cost-effectiveness of an intervention by the high intensity of infection (HII) years averted per US$ spent over the course of the intervention. As recommended by WHO a discount rate of 3% per annum was applied to both the costs and effects [37].

As well as considering specific vaccination costs, we calculate a critical vaccination cost, which is the cost per person vaccinated, relative to MDA per person treated, to achieve the same cost efficacy as MDA. This can be considered to be a maximum price one would be willing to pay for vaccination, on the assumption that cost is the only deciding factor. In practice it may not be as simple as this.

## 3 Results

### 3.1 Effectiveness of control strategies

In this section, for each treatment strategy, we evaluate the probability of achieving the WHO goals 15 years post initiation of control. We find that the outcome depends on the transmission intensity (the magnitude of the basic reproductive number, R_0_), the goal and treatment strategy (vaccine only with different durations of protection, MDA only, and vaccine plus MDA).

**Fig. 1:**
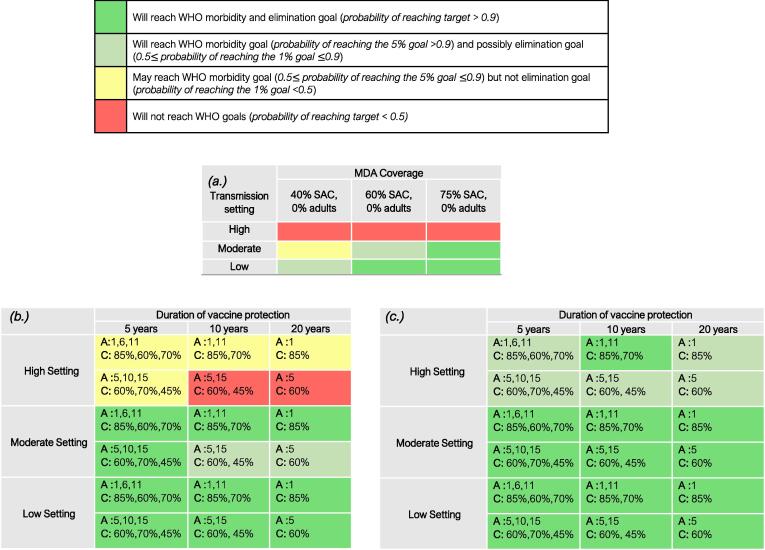
Projected outcomes for *S. mansoni* employing various control strategies as judged by reference to achieving the WHO guidelines for control (5% morbidity control and 1% elimination as a public health problem). Results for different durations of vaccine protection and vaccine coverage levels in low (<10% baseline prevalence among SAC), moderate (10–50% baseline prevalence among SAC) and high (≥50% baseline prevalence among SAC) settings. (a.) represents results for MDA only with different coverage levels among SAC (assuming random compliance at each round of treatment). (b.) shows results for vaccine only with different durations of protection and coverage levels. (c.) shows results for MDA plus immunization where the WHO target of 75% SAC coverage is achieved. In (b) and (c) The first-row values (denoted by A:) represent the cohort ages vaccinated, while the second-row values (denoted by C:) represent the corresponding coverage levels. Results shown are generated by the ICL model.

In low transmission settings, the WHO goals can be achieved for all treatment strategies (Fig. 1, S.1-S.4 and Figure S.1). For MDA alone, SAC coverage as low as 40% can achieve the morbidity control goal and potentially the EPHP goal (Fig. 1(a.)).

In moderate transmission settings, using the recommended target coverage of 75% for SAC, the SAC morbidity goal and EPHP goal can be achieved within 15 years of the initiation of MDA (Fig. 1(a.) and Figure S.2). When cohort immunization is considered alone, the outcome depends on the duration of vaccine protection and coverage levels achieved. A vaccine that provides 5 years of protection can achieve both goals within 15 years of the initiation of treatment (Fig. 1(b.) and Figure S.3).

**Fig. 2:**
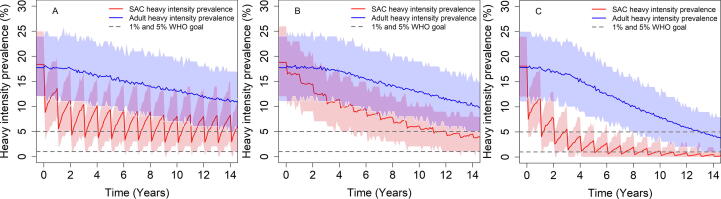
Prevalence of heavy-intensity infections in school-aged children (SAC) and adults for high transmission settings. Graph (A) represents MDA only scenario where MDA is given to 75% of SAC. Graph (B) represents cohort immunization only scenario where the duration of vaccine protection is 10 years, vaccinating 1 and 11-year-old with coverage of 85% and 70% respectively. Graph (C) represents SAC MDA + cohort immunization. Duration of vaccine protection is 10 years treating 1 and 11-year old with a coverage of 85% and 70% respectively. MDA to 75% of SAC. Results shown are generated by the ICL model.

In high transmission settings MDA alone (with the recommended 75% coverage) cannot achieve the WHO goals (Fig. 2(A) and Fig. 1(a.)). Cohort vaccination can achieve the morbidity control goal, but it is very unlikely to achieve the EPHP goal (Fig. 2(B) and Fig. 1(b.)). We can modify this outcome by immunizing across bands of age classes (e.g. including adults). However, this may risk a high frequency of adverse effects due to past or present infection in vaccinated individuals. The best strategy in these circumstances is intensive MDA targeted at SAC combined with immunization. Treating 75% of SAC with MDA and vaccinating 1 and 11-year old can achieve the morbidity control goal with probability of 100% and the EPHP with a probability of nearly 90% within 15 years (Fig. 2(C) and Fig. 1(c.)).

Alternatively, cohort immunization with a catch-up campaign and MDA, or immunization of SAC/community every two years can achieve the WHO goals within 15 years of treatment.

### 3.2 Cost-effectiveness using infant starting immunization programs

The simulation results presented in the previous section show that the different treatment strategies can generate similar results in terms of achieving the WHO goals. Logistical, programmatic and cost issues will therefore play an important role in decision making about which strategy to adopt and whether to proceed in vaccine development.

**Fig. 3:**
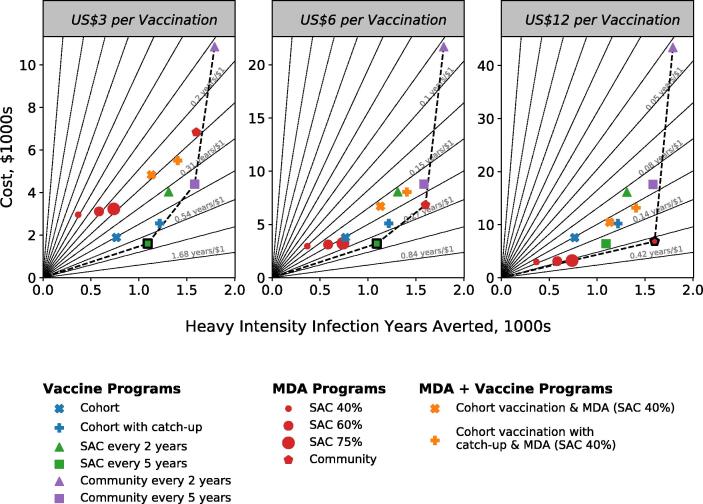
Incremental cost-effectiveness ratio (ICER) diagrams, comparing costs to HII years averted across different interventions, in the high-transmission setting over 15 years, for a vaccine that confers 15 years of protection. Cohort vaccination starts at age 1. Left, middle and right plots compare interventions where vaccination costs $3, $6 and $12 per full course of vaccination, respectively. Radial gridlines indicate programs of equal cost-efficacy. In each ICER diagram, the marker with the bold outline indicates the strategy that is most cost-effective. The dashed line shows the location of the efficient frontier, which estimates the maximum achievable HII years averted for a given cost. Results shown are generated by the UOW model.

Fig. 3 shows how the cost-effectiveness of each intervention strategy compares in the high transmission setting, measured after 15 years of intervention, for a vaccine that confers 10 years of protection. We find that community-wide MDA is more cost-effective than school-based MDA and is the most cost-effective strategy when vaccination costs US$12. When vaccination costs less than US$6, vaccination of SAC every 5 years is the most cost-effective strategy for all durations of protection. Cohort vaccination is more cost-effective than school-based MDA when vaccination costs US$6 or less. These patterns are repeated for vaccines with durations of 5 and 20 years (Figure S.8).

For low and moderate transmission settings (Figure S.6 and Figure S.7), school-based MDA with 75% coverage is the most cost-effective intervention when the cost of vaccination is US$6 or over, for all vaccine protection durations. When vaccination costs US$3 and under, then vaccination of SAC every 5 years is the most cost-effective intervention for all vaccine protection durations.

Other general trends we observe are: (i) In low and moderate transmission settings, increasing coverage of children in SAC MDA is more cost-effective than broadening the intervention to include adults without increasing coverage in children, although community-wide MDA is able to avert more high-intensity infections and reach the goal faster. (ii) A catch-up campaign becomes increasingly beneficial for cohort vaccination as the duration of vaccine protection increases. (iii) Community vaccination averts the most heavy intensity infections, but they are the most costly interventions. The longer the duration of vaccine protection, the faster the intervention is able to break transmission, shown by the decreasing trend in costs (purple triangles). (iv) With a vaccine that confers 20 years of protection, cohort vaccination with a catch-up campaign becomes close in terms of performance and cost to the vaccination of SAC every 5 years. (v) Combining MDA with vaccination increases the HII years averted but decreases the cost-effectiveness of the strategy, because children are treated/vaccinated multiple times, often unnecessarily. A more targeted approach to chemotherapy with PZQ may offer higher cost-effectiveness when used in conjunction with vaccination if the costs of targeted delivery are not too high.

**Fig. 4:**
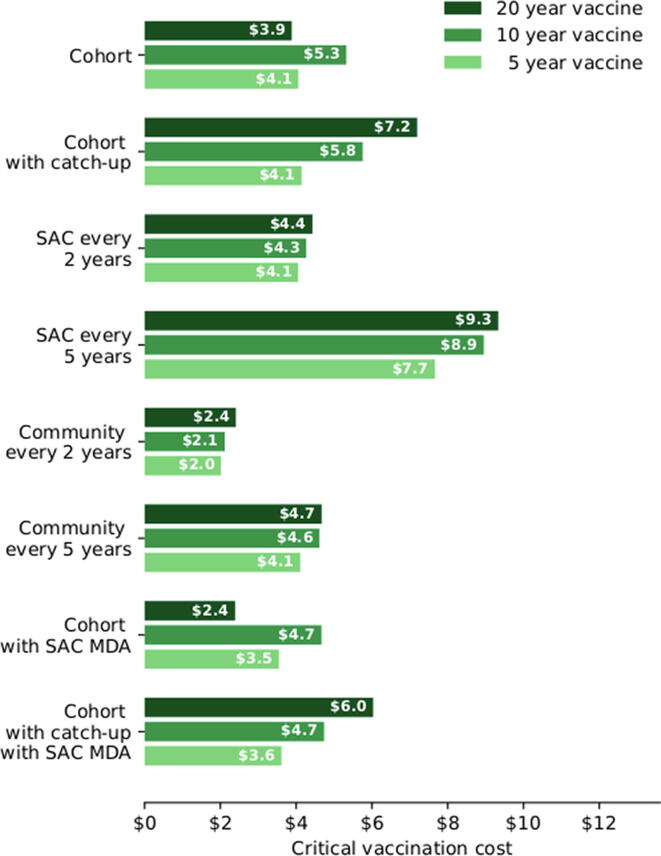
Critical vaccination costs for each intervention (relative to the most cost-effective MDA scenario) in the high-transmission setting. Cohort vaccination starts at age 1. Results shown are generated by the UOW model.

In Fig. 4, for high transmission settings we calculate a critical vaccination cost for each intervention. This is the cost for a course of vaccination that leads to the same cost-effectiveness figure as community-wide MDA. This is in effect, a maximum price that should be paid for vaccination, should cost be the only deciding factor.

Increasing the duration of protection lowers the critical vaccination costs, except in cohort strategies that do not have a catch-up campaign. This trend highlights the importance of a catch-up campaign for vaccines with a long duration of protection when employing a cohort program. The analyses suggest that even with a vaccine which confers a relatively short duration of protection, such as 5 years, it can be cost effective to pay up to US$7.60 per vaccination (or short course of vaccination). The critical vaccination costs are lower in low and medium transmission settings because re-infection after chemotherapy is slower, reducing the benefits of vaccination relative to MDA.

### 3.3 Comparison of strategies using school-based immunization programs

In Figures S.9-S.12 we compare the cost-effectiveness of immunization strategies where immunization begins at age 5, in low, moderate and high transmission settings. The results are qualitatively very similar to those above where we compared immunization strategies that start at age 1 (Figures 3, S.6 and S.7). In contrast to a cohort immunization strategy that starts with 1-year old infants, there is no disadvantage employing a vaccine with a 20-year duration of protection, in not having a catch-up campaign in terms of cost effectiveness (Figure S8). However, as well as lower costs, the heavy intensity infection years averted are greatly reduced in the absence of the catch-up. Starting cohort strategies at age 5 improves the cost-effectiveness as fewer immunizations are required and children under 5 do not harbor significant parasite burdens.

## 4 Discussion

Our analyses suggest that the current program of intensive Mass Drug Administration (MDA) using PZQ and targeted at school-aged children (SAC) is unlikely to achieve the WHO goals over a 15-year time-span, in high transmission settings. The expected availability of a PZQ pediatric indication – expected sometime in the first years of the next decade - will improve the situation in terms of making it possible to treat the very young. However, it will not address in any significant fashion the limitations of MDA without achieving much higher coverage levels than those that pertain in most endemic regions at present. It is also important to bear in mind that the repeated use of PZQ at high coverage levels may lead to the emergence and spread of drug-resistant parasites, rapidly negating the efficacy of this drug. It is therefore important that other forms of control, in particular immunization, are actively explored.

Of the scenarios examined, high intensity programs, such as community wide MDA or community wide immunization, have the greatest impact on the burden of heavy intensity infection in the community, and have the greatest chance of achieving the WHO goals of morbidity control or EPHP. For strategies targeted at school-aged children, immunization of age-cohorts combined with MDA and a catch-up campaign have the highest probabilities of meeting the WHO goals. However, in low to moderate-transmission settings less intense strategies can also be successful. Immunization is therefore likely to have a clear role to play in the control of schistosomiasis, in high-transmission setting and if the long-term goal is to break the transmission cycle, provided the current candidate vaccine is successfully navigated through the development cycle.

The most cost-effective strategy is highly dependent on the transmission-setting, the duration of vaccine protection, the level of coverage that can be obtained and the cost of the treatments. It is also dependent on other factors such as whether the vaccine can be safely administered to those who already have experience of infection or who are currently infected. If the vaccination induces adverse events in such individuals, then vaccination will have to be restricted to infants in a cohort program with no catchup activity. Another factor of importance is the time scale of analysis and the assumption that the greatest proportion of PZQ currently employed in MDA programs continues to be donated free of charge such that the only cost is that of delivery and administration to individuals.

The following discussion assumes that (i) both adverse events post immunization are not common, and (ii) the drug will continue to be donated for the foreseeable future. In low and moderate transmission settings MDA is the most cost-effective strategy over the times scales considered, with high-coverage of school age children being the best of all the strategies considered. In high-transmission settings, and for vaccines that offer 5 years or more protection, immunization of all school-aged children every 5 years is the most cost-effective strategy simulated. It results in a larger decline in heavy intensity infection than school-based MDA alone. This strategy would remain the most cost-effective strategy in high transmission settings for a vaccine that costs up to US$9.20 per full vaccination (including delivery and vaccine costs). The analysis we have performed assumes that vaccination programs begin without any prior control, but in reality, the most likely scenario is that vaccination in area will commence after having received rounds of MDA. For a given baseline transmission intensity, this will lower the cost-effectiveness estimates. Further analysis, with good quality data from field studies to inform us of the likely epidemiological settings, will improve our results.

It should be noted here that the total cost for full clinical development of the candidate (through first into humans, and through phase I to III trials) must not be a deterrent to the current major vaccine manufacturing companies. There also needs to be the necessary infrastructure to deliver the vaccine to the appropriate age-groups at appropriate intervals given that the properties of the candidate are not known at present, in terms of storage and shelf-life. Also, with respect to catch up campaigns, an infrastructure in health delivery must be established to deliver immunization across a wide band of age classes.

The efficacy of the vaccine in humans is obviously a very important consideration. In this study we have modelled the effect of a vaccine that offers perfect protection from infection, which is very close to the effect of the Sm p80 vaccine in the baboon animal model, where the multiple modes of action each have efficacies greater than 90%, which when combined give a near perfect vaccine [27], [28]. At this early stage of development, it is too soon to tell if this level of efficacy will be matched in human hosts. However, our simulated results suggest that even a partially efficacious vaccine that reduces fecundity by 75% and worm establishment by 75% produces similar results to the ideal vaccine, due to the multiplicative effect of the vaccine characteristics (see Fig S12). However, an inability to achieve an adequate efficacy, a good duration of protection and affordable pricing points would necessitate a re-evaluation of whether to proceed with vaccine development.

The choice of whether or not to proceed with clinical development of a candidate vaccine is also dependent on a set of contextual factors and conditions. For example,: (i) the desired goal (morbidity control, EPHP, full interruption of transmission) will dictate the need for a vaccine where transmission interruption is unlikely to be achieved without immunization, (ii) the proportion of remaining high transmission regions over all endemic settings where a vaccine is most cost effective and (iii) the greater the risk of PZQ resistance and hybridization of *Schistosoma* species, the greater the need for a vaccine to prevent infection in humans.

If the desired goals combined with a broad risk assessment, call for a vaccine to be part of the control strategy and that the required vaccine characteristics can be achieved, much can be learnt from the role out of the meningococcal bacterial vaccines in resource poor settings. The approach adopted for the MenAfriVac vaccine [38], [39], was one where the prospective producer shares the risk with a public health player (e.g. aid agency, UN agency, philanthropic organization) that provides some form of financial incentive.

Any manufacturer of the vaccine will face risks in a number of areas. These include (i) the ability of the development program to confirm the high effectiveness in humans that has been demonstrated in non-human primates, (ii) uncertainty in the availability of a suitable adjuvant, (iii) the potential impact of PZQ and previous infection on vaccine efficacy and/or the occurrence of adverse events, (iv) the absence of clearly defined correlates of protection that could make the approval process longer and more expensive and (v) the achievable cost of goods and hence the price of the vaccine whose level affects the overall viability of the initiative.

If the stages of first into humans and the phase I clinical trials in humans go well, the early identification of a suitable manufacturer (possibly from the emerging economies) who is prepared to take the risks outlined above is crucial. Areas with endemic *S mansoni* infection are primarily in Africa and clinical trials will need to be run in this region. Partnering the potential manufacturer with relevant bodies in Africa will be crucial to success as well illustrated by the recent development and trials of a Plasmodium falciparum vaccine (RTS, S) [40].

## 5 Conclusions

Our analyses suggest that MDA alone can achieve the WHO goals in low to moderate but not in high transmission settings. The simulation studies clearly indicate that a vaccine is needed to both reduce the probability of reinfection and eliminate transmission within a reasonable time frame. The value of the vaccine depends on, the duration of vaccine protection, the public health goal (reduction in the prevalence of infection or the prevalence of heavy-intensity infection) and the cost of the treatment. In high transmission settings, vaccination alone may achieve the morbidity control goal, but integrated MDA plus vaccination is required to achieve the EPHP goal. In these settings, vaccination can be more cost-effective than the current MDA programmes.

## CRediT authorship contribution statement

**Klodeta Kura:** Conceptualization, Formal analysis, Investigation, Methodology, Writing - original draft. **Benjamin S. Collyer:** Conceptualization, Formal analysis, Investigation, Methodology, Writing - review & editing. **Jaspreet Toor:** Conceptualization, Writing - review & editing. **James E. Truscott:** Methodology, Writing - review & editing. **T. Deirdre Hollingsworth:** Conceptualization, Methodology, Writing - review & editing. **Matt J. Keeling:** Conceptualization, Methodology, Writing - review & editing. **Roy M. Anderson:** Conceptualization, Methodology, Writing - review & editing.

## Declaration of Competing Interest

The authors declare that they have no known competing financial interests or personal relationships that could have appeared to influence the work reported in this paper.
