# Supplementary material for: Policy implications of the potential use of a novel vaccine to prevent infection with Schistosoma mansoni with or without mass drug administration
Source: Vaccine. 2020 Jun 9;38(28):4379–86. doi: 10.1016/j.vaccine.2020.04.078 (PMC7273196; doi:10.1016/j.vaccine.2020.04.078)
Supplement: Supplementary data 1 [file mmc1.docx]

**Supplementary Information**

**Table S1: Projected outcomes for *S. mansoni* employing various control strategies as judged by reference to achieving the WHO guidelines for control (5% morbidity control and 1% EPHP).** Results for cohort immunization + vaccine catch-up campaign (immunizing across a broad range of age classes).

|  | **Duration of vaccine protection** | | |
| --- | --- | --- | --- |
|  | **5 years** | **10 years** | **20 years** |
| **High Setting** | **Vaccinate ages:**1,6,11  **Coverage:** 85%,60%,70% | **Vaccinate ages :**1,11  **Coverage:** 85%,70% | **Vaccinate ages :**1  **Coverage:** 85% |
|  | **Vaccinate ages :**5,10,15  **Coverage:** 60%,70%,45% | **Vaccinate ages :**5,15  **Coverage:** 60%, 45% | **Vaccinate ages :**5  **Coverage:** 60% |
| **Moderate Setting** | **Vaccinate ages :**1,6,11  **C:** 85%,60%,70% | **Vaccinate ages :**1,11  **C:** 85%,70% | **Vaccinate ages :**1  **Coverage:** 85% |
|  | **Vaccinate ages :**5,10,15  **Coverage:** 60%,70%,45% | **Vaccinate ages :**5,15  **Coverage:** 60%, 45% | **Vaccinate ages :**5  **C:** 60% |
| **Low Setting** | **Vaccinate ages :**1,6,11  **Coverage:** 85%,60%,70% | **Vaccinate ages :**1,11  **Coverage:** 85%,70% | **Vaccinate ages :**1  **Coverage:** 85% |
|  | **Vaccinate ages :**5,10,15  **Coverage:** 60%,70%,45% | **Vaccinate ages :**5,15  **Coverage:** 60%, 45% | **Vaccinate ages :**5  **Coverage:** 60% |

**Table S2: Projected outcomes for *S. mansoni* employing various control strategies as judged by reference to achieving the WHO guidelines for control (5% morbidity control and 1% EPHP).** Results for cohort immunization + vaccine catch-up campaign + SAC MDA (40% coverage).

|  | **Duration of vaccine protection** | | |
| --- | --- | --- | --- |
|  | **5 years** | **10 years** | **20 years** |
| **High Setting** | **Vaccinate ages:**1,6,11  **Coverage:** 85%,60%,70% | **Vaccinate ages :**1,11  **Coverage:** 85%,70% | **Vaccinate ages :**1  **Coverage:** 85% |
|  | **Vaccinate ages :**5,10,15  **Coverage:** 60%,70%,45% | **Vaccinate ages :**5,15  **Coverage:** 60%, 45% | **Vaccinate ages :**5  **Coverage:** 60% |
| **Moderate Setting** | **Vaccinate ages :**1,6,11  **C:** 85%,60%,70% | **Vaccinate ages :**1,11  **C:** 85%,70% | **Vaccinate ages :**1  **Coverage:** 85% |
|  | **Vaccinate ages :**5,10,15  **Coverage:** 60%,70%,45% | **Vaccinate ages :**5,15  **Coverage:** 60%, 45% | **Vaccinate ages :**5  **C:** 60% |
| **Low Setting** | **Vaccinate ages :**1,6,11  **Coverage:** 85%,60%,70% | **Vaccinate ages :**1,11  **Coverage:** 85%,70% | **Vaccinate ages :**1  **Coverage:** 85% |
|  | **Vaccinate ages :**5,10,15  **Coverage:** 60%,70%,45% | **Vaccinate ages :**5,15  **Coverage:** 60%, 45% | **Vaccinate ages :**5  **Coverage:** 60% |

**Table S3: Projected outcomes for *S. mansoni* employing various control strategies as judged by reference to achieving the WHO guidelines for control (5% morbidity control and 1% EPHP).** Results for immunization of SAC.

|  |  | **Duration of vaccine protection** | | |
| --- | --- | --- | --- | --- |
|  | **Frequency** | **5 years** | **10 years** | **20 years** |
| **High Setting** | Every year |  |  |  |
|  | Every 2 years |  |  |  |
|  | Every 5 years |  |  |  |
| **Moderate Setting** | Every year |  |  |  |
|  | Every 2 years |  |  |  |
|  | Every 5 years |  |  |  |
| **Low Setting** | Every year |  |  |  |
|  | Every 2 years |  |  |  |
|  | Every 5 years |  |  |  |

**Table S4: Projected outcomes for *S. mansoni* employing various control strategies as judged by reference to achieving the WHO guidelines for control (5% morbidity control and 1% EPHP).** Results for the immunization of the entire community for different vaccine protection durations.

|  |  | **Duration of vaccine protection** | | |
| --- | --- | --- | --- | --- |
|  | **Frequency** | **5 years** | **10 years** | **20 years** |
| **High Setting** | Every year |  |  |  |
|  | Every 2 years |  |  |  |
|  | Every 5 years |  |  |  |
| **Moderate Setting** | Every year |  |  |  |
|  | Every 2 years |  |  |  |
|  | Every 5 years |  |  |  |
| **Low Setting** | Every year |  |  |  |
|  | Every 2 years |  |  |  |
|  | Every 5 years |  |  |  |

**Table S5: Economic and financial costs for MDA delivery, obtained from online WHO regression tool** [30]**. Financial costs are the monetary expenditure directly related to the activity. Economic costs include costs associated with Ministry of Health buildings and time, as well as the value associated with volunteers’ time. * - includes $0.08 per person for the** **opportunity costs related to the community volunteers who distribute the drugs which are not included with the WHO regression tool** [31]**.**

| **Economic costs** | | | **Financial costs** | | |
| --- | --- | --- | --- | --- | --- |
| **Cost** | **School-based Delivery** | **Community-wide Delivery** | **School-based Delivery** | **Community-wide Delivery** | **References** |
| Delivery cost (per person targeted) | $0.75 | $0.50* | $0.31 | $0.34 | [30] |
| PZQ  tablets | 2.5 x $0.08 per child | 2.5 x $0.08 per child  3.5 x $0.08 per adult | Donated | Donated | [32], [33] |

| 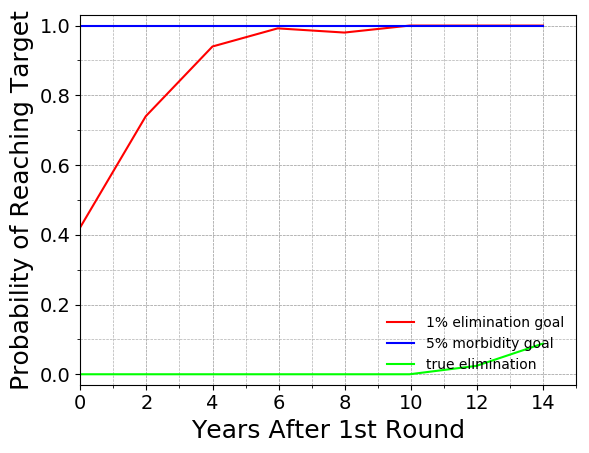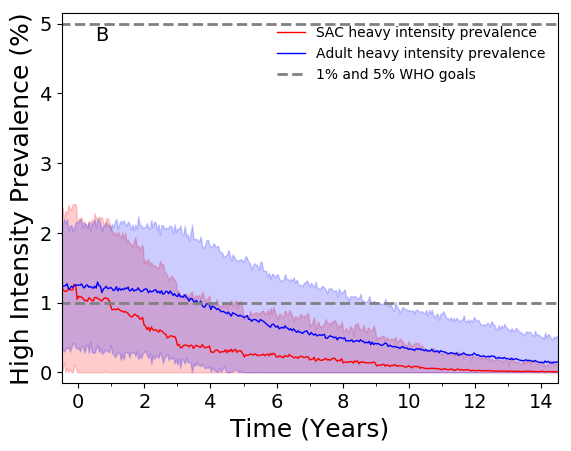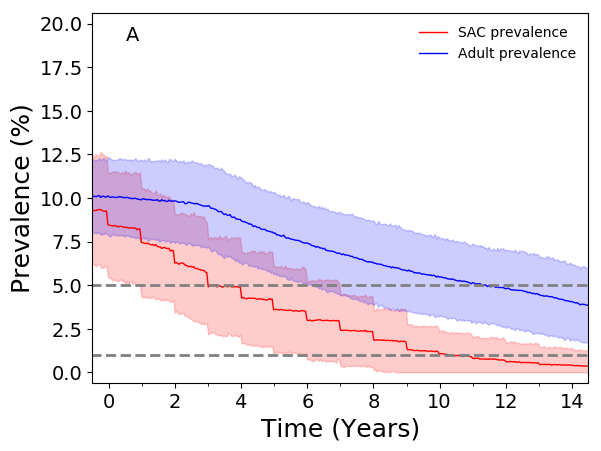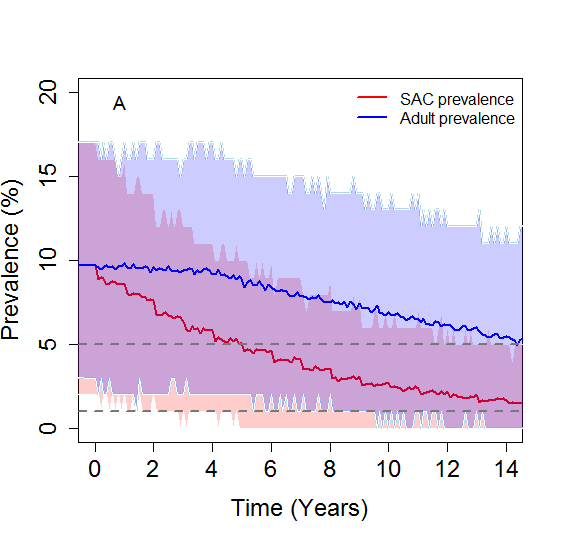 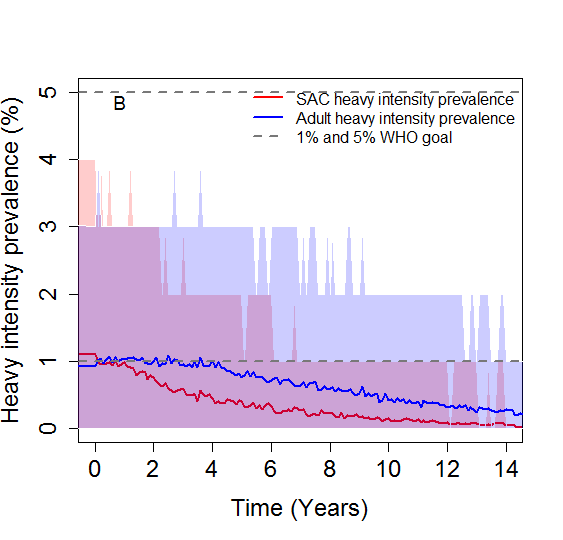 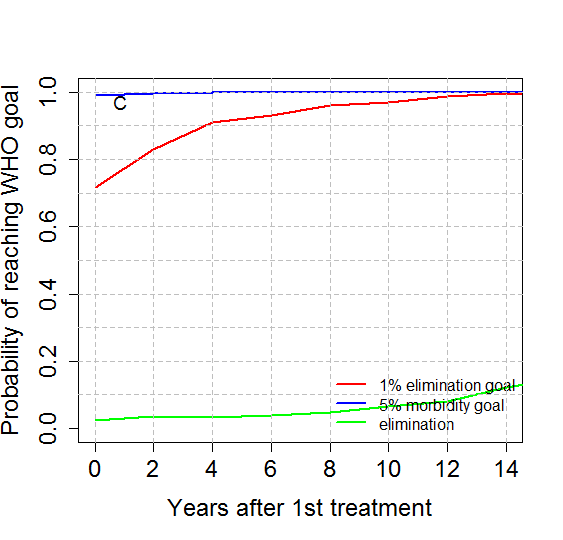 |
| --- |
| **Figure S.1: Cohort Immunization:** **Imperial College London (top row) and Warwick (second row) model scenarios showing the prevalence of infection (A), prevalence of heavy-intensity infections (B) in school-aged children (SAC) and adults for low baseline settings.** Duration of protection is 10 years vaccinating 1 and 11-year olds with a coverage of 85 % and 70% respectively. The WHO goals can be achieved within 5 years of treatment. (C) represents the probability of reaching the WHO goals by year 15 prior initiation of immunization. |

| 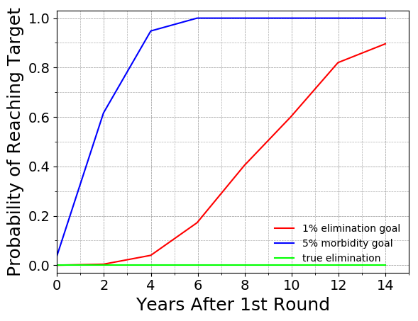*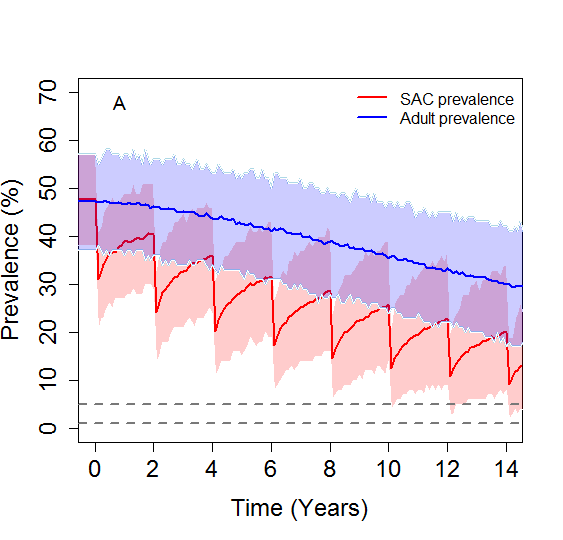*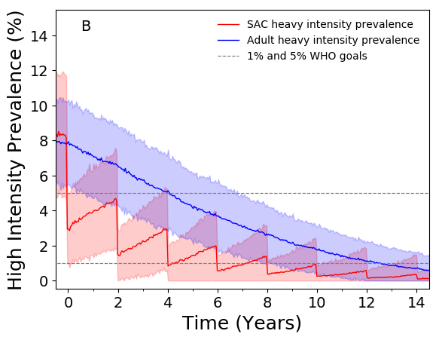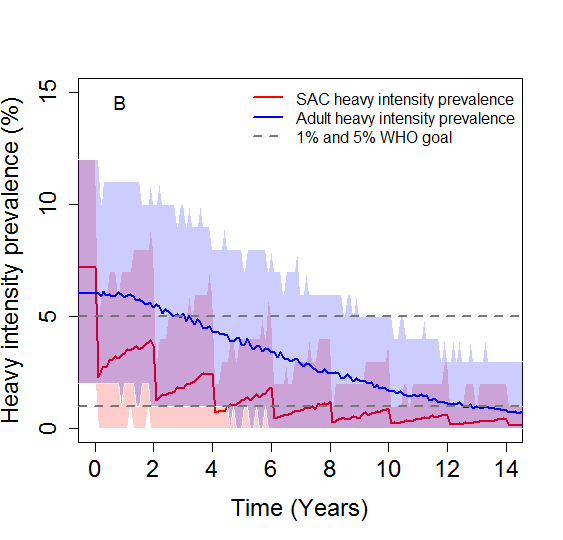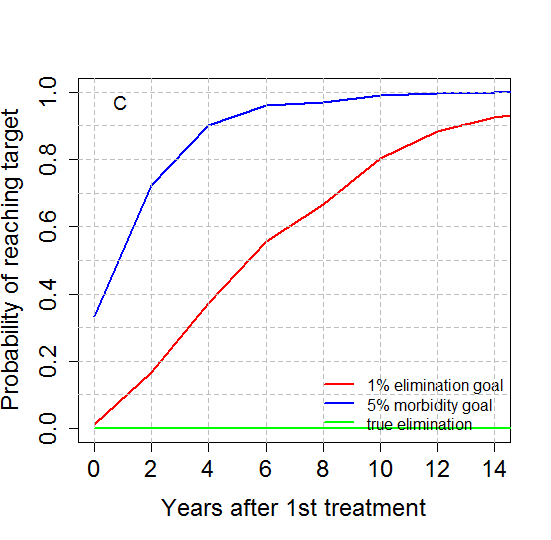 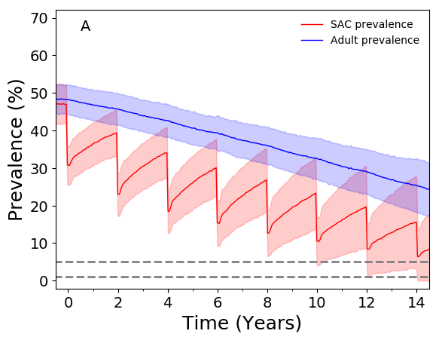 |
| --- |
| **Figure S.2: SAC MDA: Imperial College London (top row) and Warwick (second row) model scenarios showing the prevalence of infection (A), prevalence of heavy-intensity infections (B) in school-aged children (SAC) and adults for moderate baseline settings**. WHO target of 75% SAC coverage is assumed. Giving MDA once every two years reaches the 5% morbidity and 1% EPHP goals by year 15. Shaded areas represent the 90% credible interval. (C) represents the probability of reaching the WHO goals by year 15 prior initiation of MDA. |

| 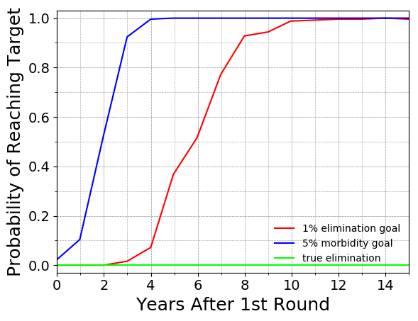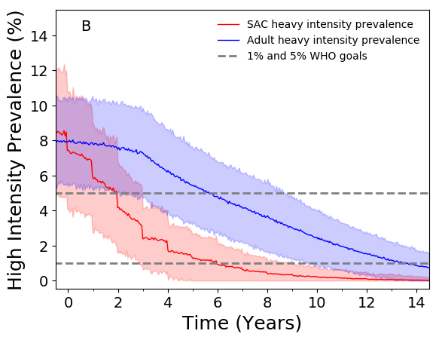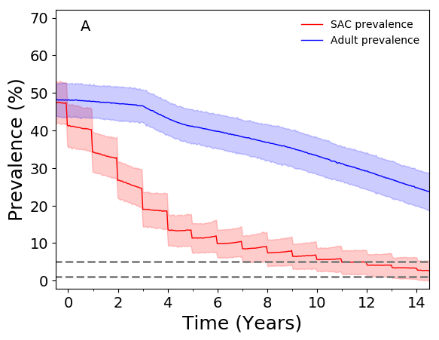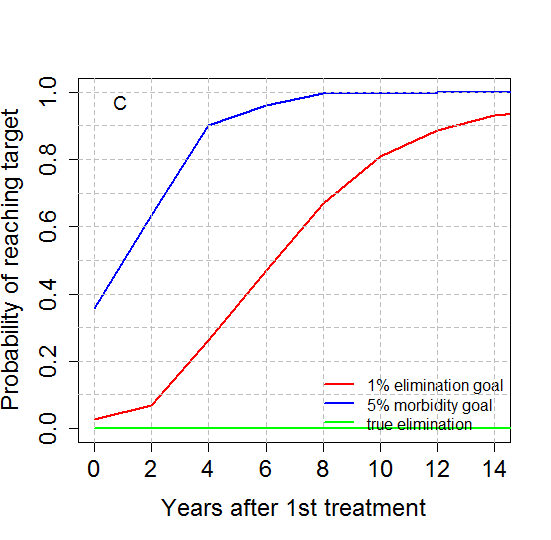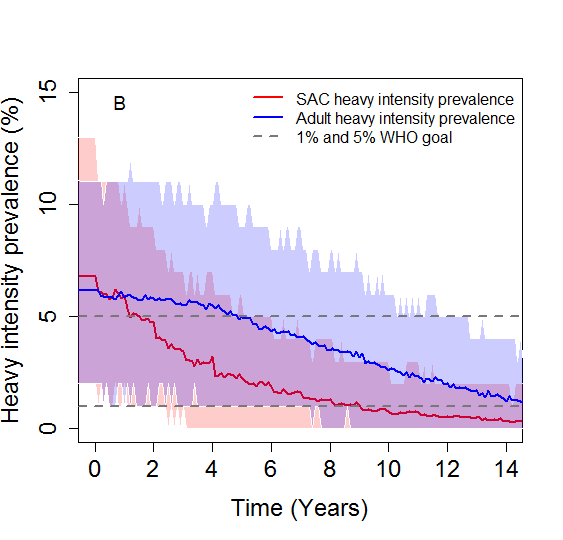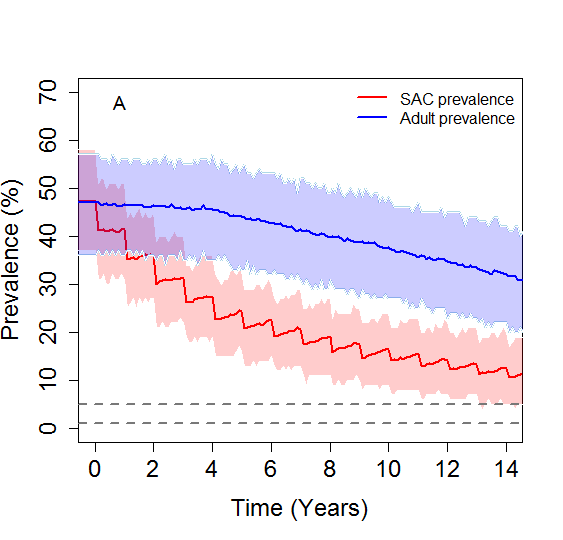 |
| --- |
| **Figure S.3: Cohort Immunization: Imperial College London (top row) and Warwick (second row) model scenarios showing the prevalence of infection (A), prevalence of heavy-intensity infections (B) in school-aged children (SAC) and adults for moderate baseline settings**. Duration of protection is 5 years treating 1, 6 and 11-year old with a coverage of 85%, 60% and 70% respectively. Probability of reaching WHO 1% and 5% goals is approximately 1. (C) represents the probability of reaching the WHO goals by year 15 prior initiation of immunization. |

| **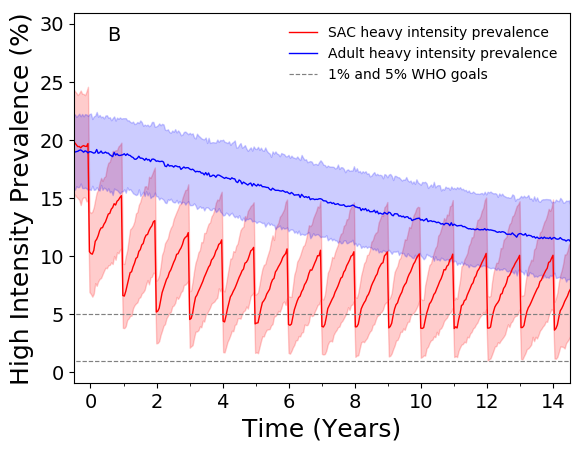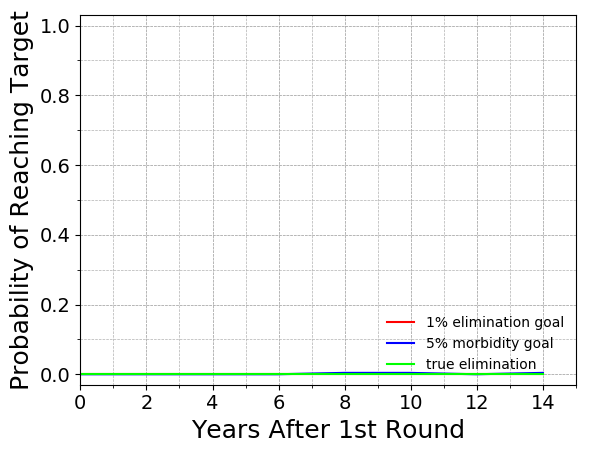**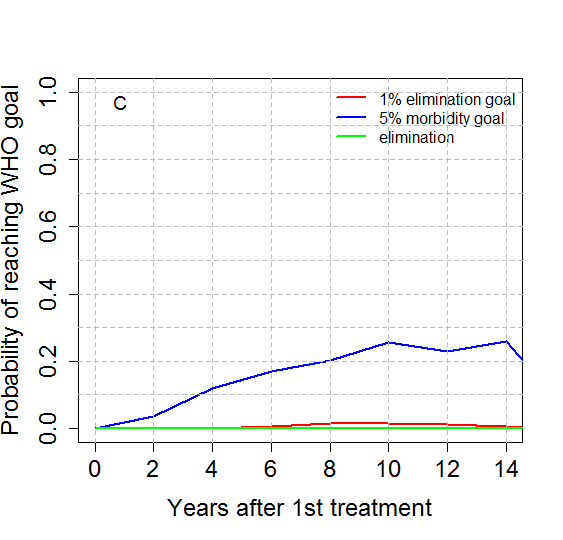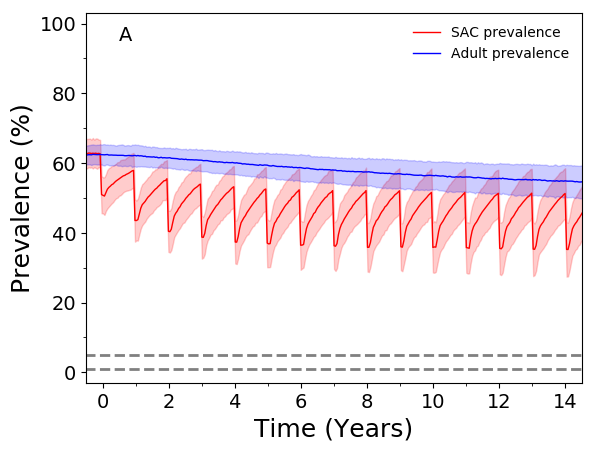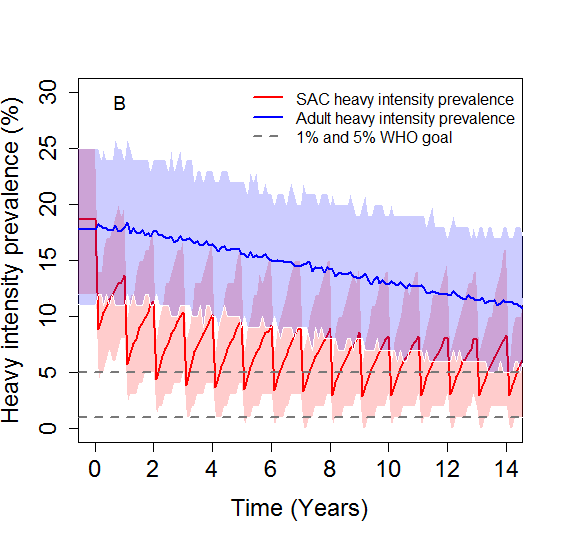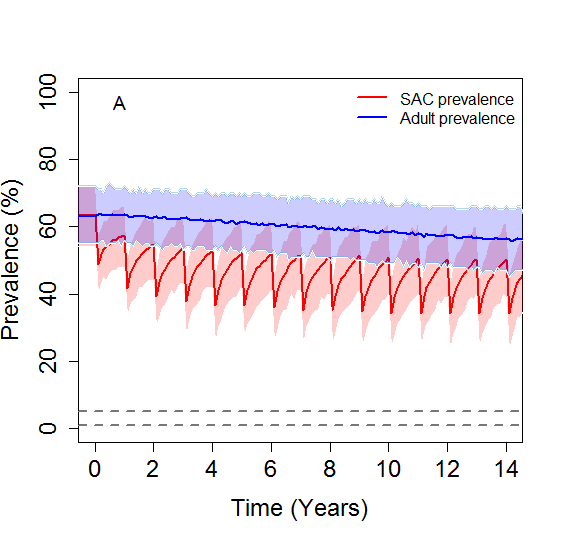 |
| --- |
| **Figure S.4: SAC MDA:** **Imperial College London (top row) and Warwick (second row) model scenarios showing the prevalence of infection (A), prevalence of heavy-intensity infections (B) in school-aged children (SAC) and adults for high baseline settings.** WHO target of 75% SAC coverage is assumed. Giving MDA once a year does not achieve the WHO goals. Shaded areas represent the 90% credible interval. (C) represents the probability of reaching the WHO goals by year 15 prior initiation of MDA. |

| 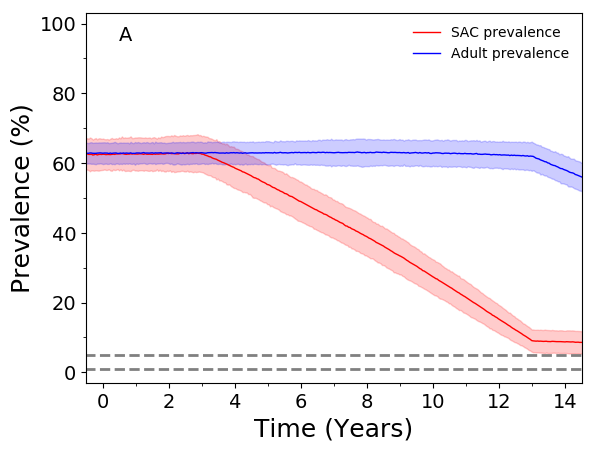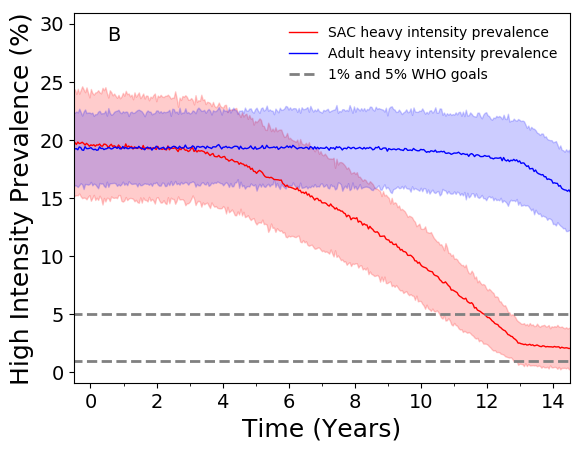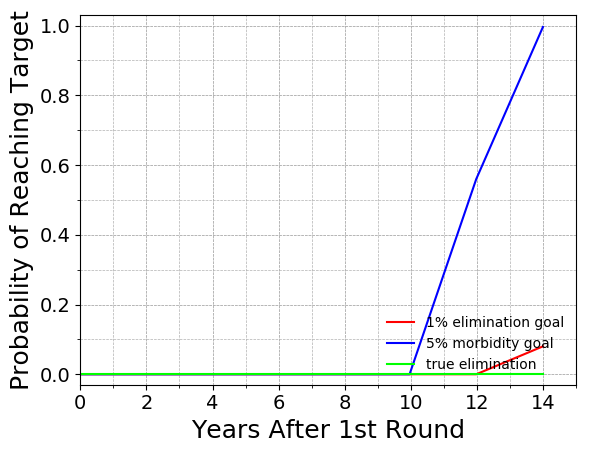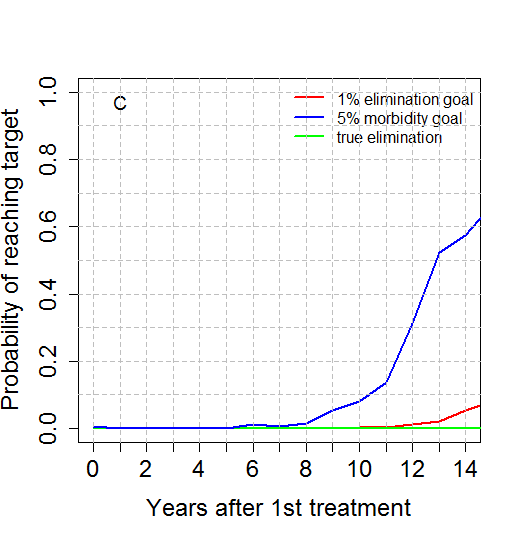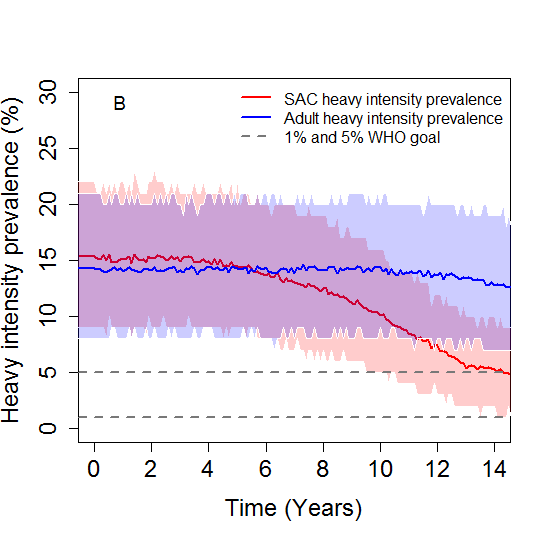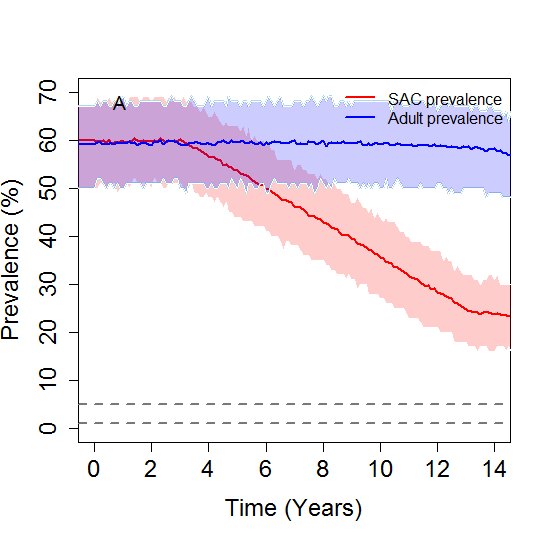 |
| --- |
| **Figure S.5: Cohort Immunization: Imperial College London (top row) and Warwick (second row) model scenarios showing the prevalence of infection (A), prevalence of heavy-intensity infections (B) in school-aged children (SAC) and adults for high baseline settings.** Duration of vaccine protection is 20 years and immunization is given annually to 85% of 1-year old. (C) represents the probability of reaching the WHO goals. |


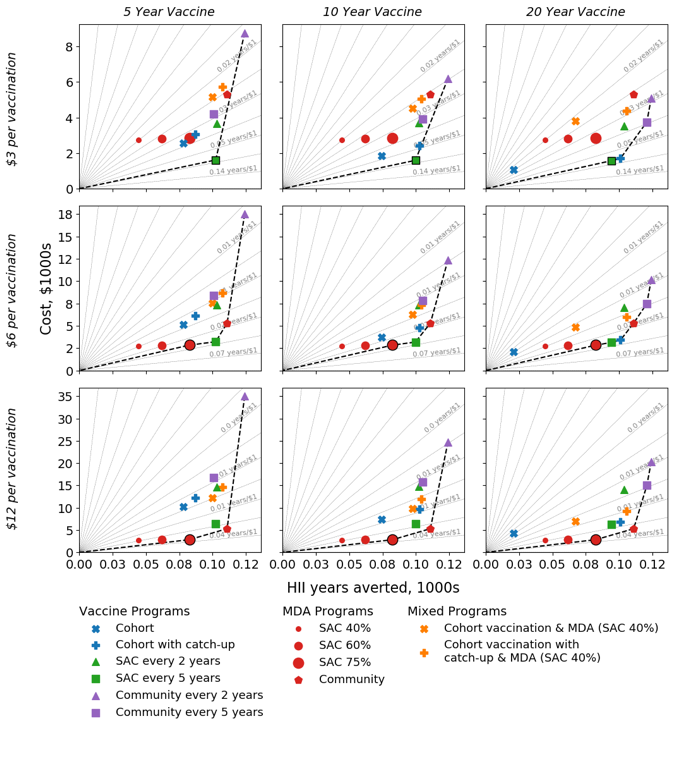


Figure S.6: Incremental cost-effectiveness ratio (ICER) diagrams, comparing costs to HII years averted across different interventions, in the low-transmission setting over 15 years for vaccines with different durations of protection. Cohort vaccination starts at age 1. Top, middle and bottom rows compare interventions where vaccination costs $3, $6 and $12 per vaccination, respectively. Left, middle and right columns compare interventions where vaccination protection lasts 5, 10 and 20 years respectively. Radial gridlines indicate programs of equal cost-efficacy. In each ICER diagram, the marker with the bold outline indicates the strategy that is most cost-effective, and the dashed line shows the location of the efficient frontier.


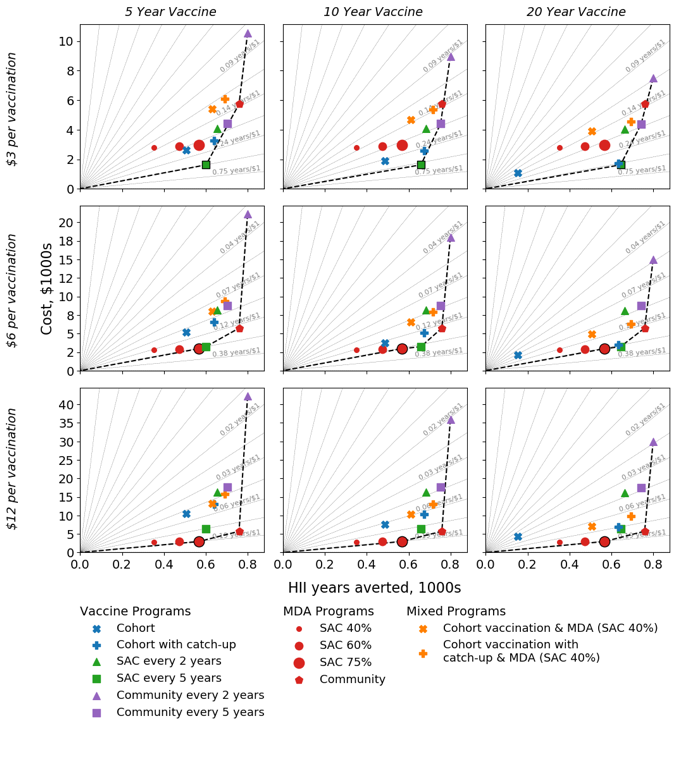


Figure S.7: Incremental cost-effectiveness ratio (ICER) diagrams, comparing costs to HII years averted across different interventions, in the moderate-transmission setting over 15 years for vaccines with different durations of protection. Cohort vaccination starts at age 1. Top, middle and bottom rows compare interventions where vaccination costs $3, $6 and $12 per vaccination, respectively. Left, middle and right columns compare interventions where vaccination protection duration lasts 5, 10 and 20 years respectively. Radial gridlines indicate programs of equal cost-efficacy. In each ICER diagram, the marker with the bold outline indicates the strategy that is most cost-effective, and the dashed line shows the location of the efficient frontier.


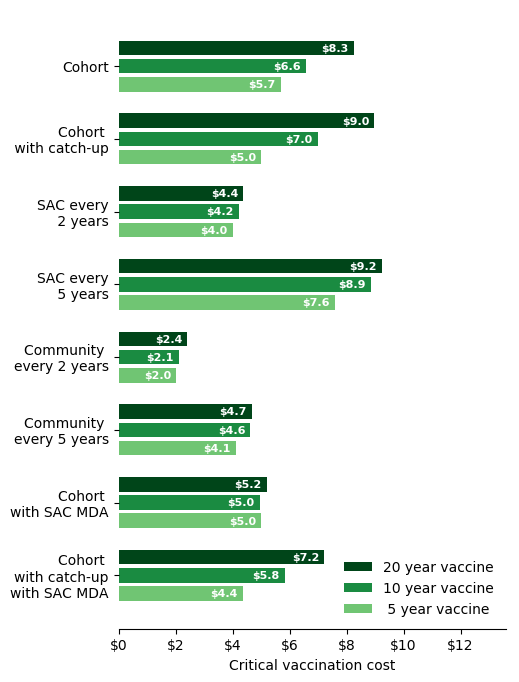


Figure S.8: Critical vaccination costs for each intervention (relative to the most cost-effective MDA scenario) in the high transmission setting. Cohort vaccination starts at age 5.


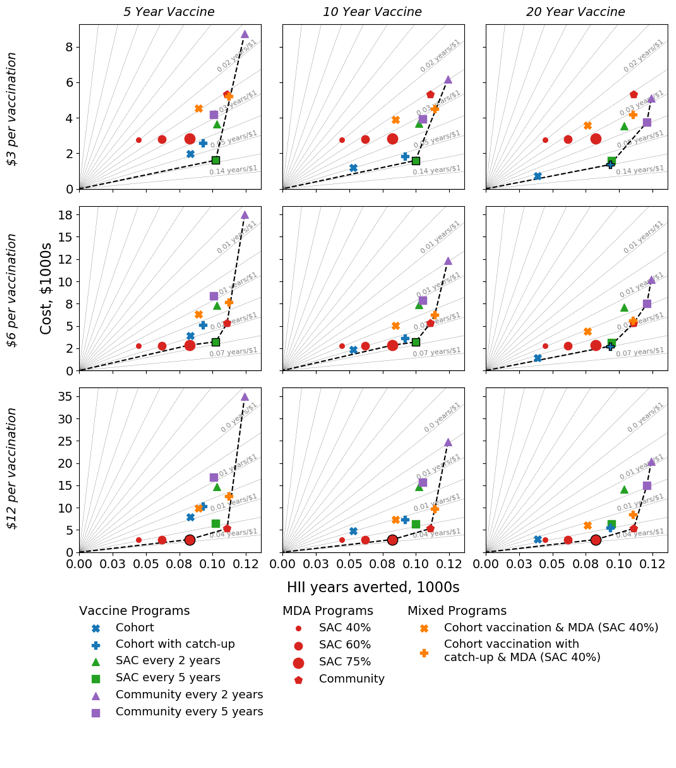


Figure S.9: Incremental cost-effectiveness ratio (ICER) diagrams, comparing costs to HII years averted across different interventions, in the low-transmission setting over 15 years for vaccines with different durations of protection. Cohort vaccination starts at age 5. Top, middle and bottom rows compare interventions where vaccination costs $3, $6 and $12 per vaccination, respectively. Left, middle and right columns compare interventions where vaccination protection lasts 5, 10 and 20 years respectively. Radial gridlines indicate programs of equal cost-efficacy. In each ICER diagram, the marker with the bold outline indicates the strategy that is most cost-effective, and the dashed line shows the location of the efficient frontier.


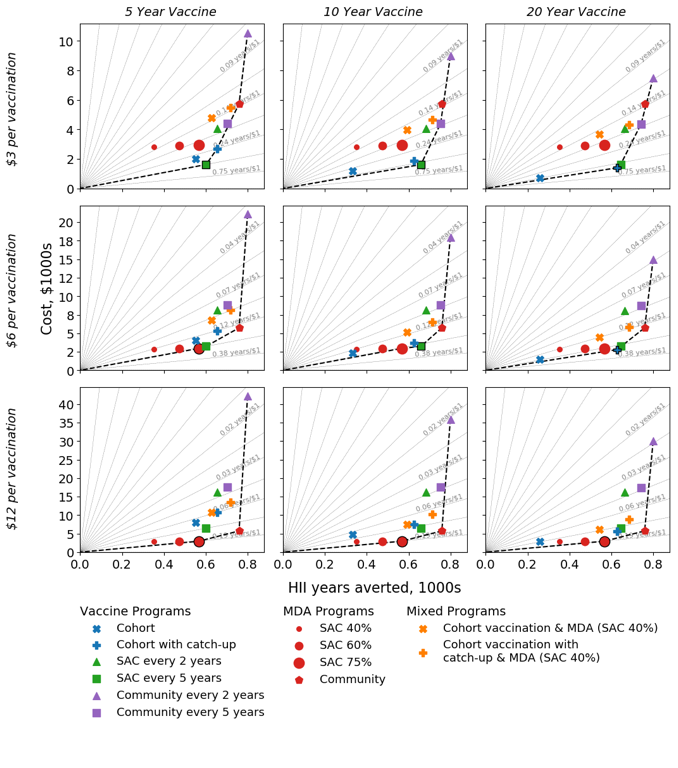


Figure S.10: Incremental cost-effectiveness ratio (ICER) diagrams, comparing costs to HII years averted across different interventions, in the moderate-transmission setting over 15 years for vaccines with different durations of protection. Cohort vaccination starts at age 5. Top, middle and bottom rows compare interventions where vaccination costs $3, $6 and $12 per vaccination, respectively. Left, middle and right columns compare interventions where vaccination protection lasts 5, 10 and 20 years respectively. Radial gridlines indicate programs of equal cost-efficacy. In each ICER diagram, the marker with the bold outline indicates the strategy that is most cost-effective, and the dashed line shows the location of the efficient frontier.


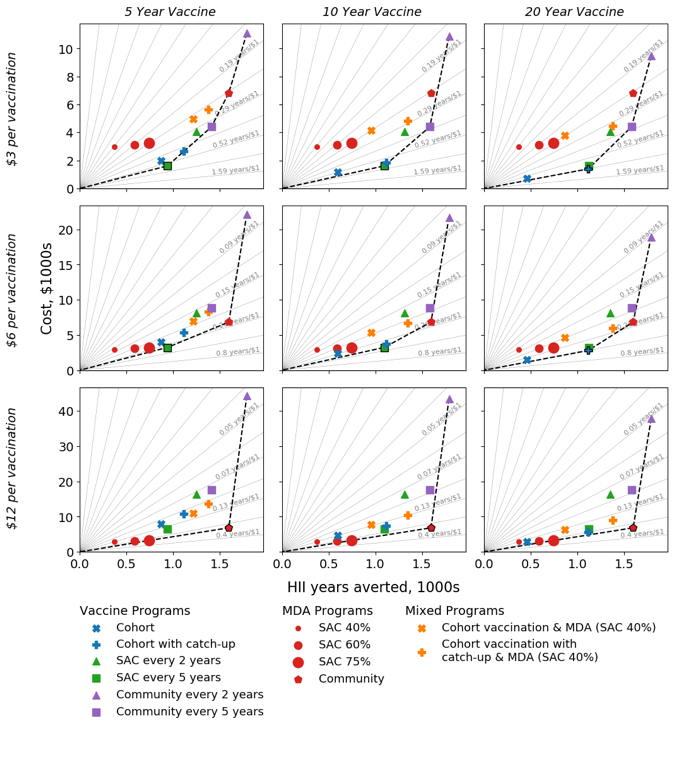


Figure S.11: Incremental cost-effectiveness ratio (ICER) diagrams, comparing costs to HII years averted across different interventions, in the high-transmission setting over 15 years for vaccines with different durations of protection. Cohort vaccination starts at age 5. Top, middle and bottom rows compare interventions where vaccination costs $3, $6 and $12 per vaccination, respectively. Left, middle and right columns compare interventions where vaccination protection lasts 5, 10 and 20 years respectively. Radial gridlines indicate programs of equal cost-efficacy. In each ICER diagram, the marker with the bold outline indicates the strategy that is most cost-effective, and the dashed line shows the location of the efficient frontier.


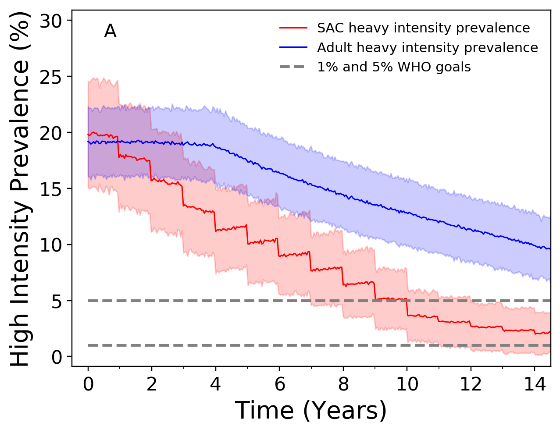

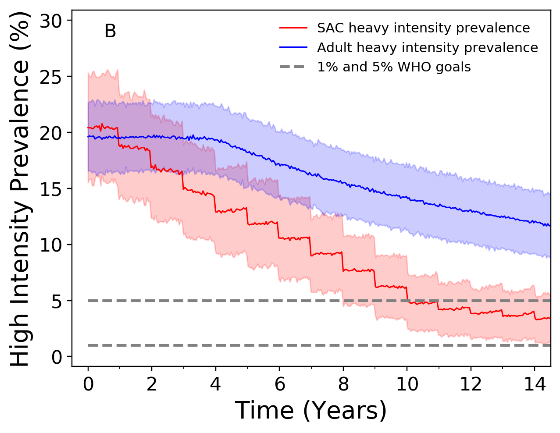


**Figure S.12: Comparison between the effect of perfect vaccine (A) and partially efficacious vaccine (B) for high baseline baseline settings**. The partially efficacious vaccine reduces female worm fecundity by 75% and worm establishment by 75%. Duration of protection for both vaccines is 10 years treating 1 and 11-year olds with a coverage of 85%, 70% respectively.
